# *N*′-(2-Chloro-5-nitro­benzyl­idene)isonicotinohydrazide

**DOI:** 10.1107/S1600536809006588

**Published:** 2009-02-28

**Authors:** Feng Zhi

**Affiliations:** a Modern Medical Research Center, Third Affiliated Hospital of Soochow University, Changzhou 213003, People’s Republic of China

## Abstract

The title compound, C_13_H_9_ClN_4_O_3_, was synthesized by the condensation reaction of 2-chloro-5-nitro­benzaldehyde with isonicotinohydrazide in a methanol solution. The mol­ecule of the compound displays a *trans* configuration with respect to the C=N and C—N bonds. The dihedral angle between the benzene and pyridine rings is 12.1 (2)°. In the crystal structure, adjacent mol­ecules are linked through inter­molecular N—H⋯O hydrogen bonds, forming dimers.

## Related literature

For Schiff base compounds, see: Fan *et al.* (2007 ▶); Kim *et al.* (2005 ▶); Nimitsiriwat *et al.* (2004 ▶). For the biological activity of Schiff base compounds, see: Chen *et al.* (1997 ▶); Ren *et al.* (2002 ▶). For similar structures, see: Mohd Lair *et al.* (2009 ▶); Fun *et al.* (2008 ▶); Yang (2008 ▶); Zhi (2008 ▶); Zhi & Yang (2007 ▶).
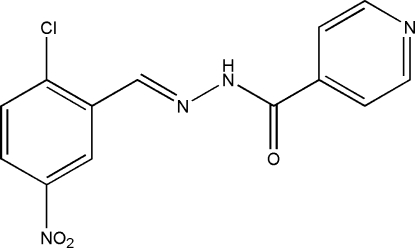


## Experimental

### 

#### Crystal data

C_13_H_9_ClN_4_O_3_
*M* *_r_* = 304.69
Tetragonal, 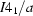

*a* = 18.586 (3) Å
*c* = 15.183 (3) Å
*V* = 5244.9 (15) Å^3^
*Z* = 16
Mo *K*α radiation
μ = 0.31 mm^−1^
*T* = 298 K
0.12 × 0.10 × 0.10 mm

#### Data collection

Bruker SMART 1000 CCD area-detector diffractometer
Absorption correction: multi-scan (*SADABS*; Sheldrick, 1996 ▶) *T*_min_ = 0.964, *T*_max_ = 0.970
16616 measured reflections
2869 independent reflections
1685 reflections with *I* > 2σ(*I*)
*R*_int_ = 0.083

#### Refinement

*R*[*F* ^2^ > 2σ(*F* ^2^)] = 0.054
*wR*(*F* ^2^) = 0.131
*S* = 1.03
2869 reflections
194 parameters
1 restraint
H atoms treated by a mixture of independent and constrained refinement
Δρ_max_ = 0.17 e Å^−3^
Δρ_min_ = −0.21 e Å^−3^

### 

Data collection: *SMART* (Bruker, 2002 ▶); cell refinement: *SAINT* (Bruker, 2002 ▶); data reduction: *SAINT*; program(s) used to solve structure: *SHELXS97* (Sheldrick, 2008 ▶); program(s) used to refine structure: *SHELXL97* (Sheldrick, 2008 ▶); molecular graphics: *SHELXTL* (Sheldrick, 2008 ▶); software used to prepare material for publication: *SHELXL97*.

## Supplementary Material

Crystal structure: contains datablocks global, I. DOI: 10.1107/S1600536809006588/hg2481sup1.cif

Structure factors: contains datablocks I. DOI: 10.1107/S1600536809006588/hg2481Isup2.hkl

Additional supplementary materials:  crystallographic information; 3D view; checkCIF report

## Figures and Tables

**Table 1:** Hydrogen-bond geometry (Å, °)

*D*—H⋯*A*	*D*—H	H⋯*A*	*D*⋯*A*	*D*—H⋯*A*
N2—H2⋯O3^i^	0.892 (10)	2.187 (13)	3.055 (3)	164 (3)

Symmetry code: (i) 
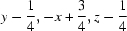
.

**Fig. 1.**
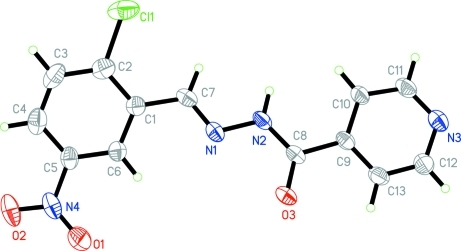
The structure of (I) at the 30% probability level.

**Fig. 2.**
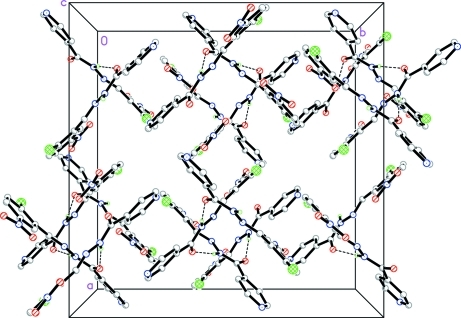
Molecular packing of (I), viewed along the b axis. Intermolecular hydrogen bonds are shown as dashed lines.

## supplementary crystallographic information

### Comment

Schiff base compounds have been widely investigated over a century (Fan *et al.*, 2007; Kim *et al.*, 2005; Nimitsiriwat *et al.*, 2004). Some of the compounds have been found to have pharmacological and antibacterial activity (Chen *et al.*, 1997; Ren *et al.*, 2002). In this paper, the crystal structure of a new Schiff base compound, (I), Fig. 1, derived from the condensation reaction of 2-chloro-5-nitrobenzaldehyde with isonicotinohydrazide is reported.

In (I), the molecular structure of the compound displays a *trans* configuration with respect to the C=N and C-N bonds. The dihedral angle between the benzene ring and the pyridine ring is 12.1 (2)°. The dihedral angle between the O1/N4/O2 plane and the benzene ring is 8.1 (2)°. All the bond lengths are within normal ranges and comparable to those in other similar compounds (Mohd Lair *et al.*, 2009; Fun *et al.*, 2008; Yang, 2008; Zhi, 2008; Zhi & Yang, 2007).

In the crystal structure, adjacent molecules are linked through intermolecular N—H···O hydrogen bonds (Table 1), forming dimers (Fig. 2).

### Experimental

2-Chloro-5-nitrobenzaldehyde (0.01 mol, 1.85 g) and isonicotinohydrazide (0.01 mol, 1.37 g) were dissolved in a methanol solution (50 ml). The mixture was stirred at room temperature to give a clear colorless solution. Crystals of the title compound were formed by gradual evaporation of the solvent for a week at room temperature.

### Refinement

The N proton H2 was located in a difference map and refined with N–H distance restrained to 0.90 (1) Å. All other H atoms were positioned geometrically [C–H = 0.93 Å] and refined using a riding model, with *U*_iso_(H) = 1.2*U*_eq_(C).

### Crystal data

**Table:**

C_13_H_9_ClN_4_O_3_	*D*_x_ = 1.543 Mg m^−^^3^
*M**_r_* = 304.69	Mo *K*α radiation, λ = 0.71073 Å
Tetragonal, *I*4_1_/*a*	Cell parameters from 1528 reflections
Hall symbol: -I 4ad	θ = 2.2–24.5°
*a* = 18.586 (3) Å	µ = 0.31 mm^−^^1^
*c* = 15.183 (3) Å	*T* = 298 K
*V* = 5244.9 (15) Å^3^	Block, colorless
*Z* = 16	0.12 × 0.10 × 0.10 mm
*F*(000) = 2496	

### Data collection

**Table:**

Bruker SMART 1000 CCD area-detector diffractometer	2869 independent reflections
Radiation source: fine-focus sealed tube	1685 reflections with *I* > 2σ(*I*)
graphite	*R*_int_ = 0.083
ω scans	θ_max_ = 27.0°, θ_min_ = 1.7°
Absorption correction: multi-scan (*SADABS*; Sheldrick, 1996)	*h* = −23→22
*T*_min_ = 0.964, *T*_max_ = 0.970	*k* = −21→23
16616 measured reflections	*l* = −19→19

### Refinement

**Table:**

Refinement on *F*^2^	Secondary atom site location: difference Fourier map
Least-squares matrix: full	Hydrogen site location: inferred from neighbouring sites
*R*[*F*^2^ > 2σ(*F*^2^)] = 0.054	H atoms treated by a mixture of independent and constrained refinement
*wR*(*F*^2^) = 0.131	*w* = 1/[σ^2^(*F*_o_^2^) + (0.0346*P*)^2^ + 2.6778*P*] where *P* = (*F*_o_^2^ + 2*F*_c_^2^)/3
*S* = 1.03	(Δ/σ)_max_ < 0.001
2869 reflections	Δρ_max_ = 0.17 e Å^−^^3^
194 parameters	Δρ_min_ = −0.21 e Å^−^^3^
1 restraint	Extinction correction: *SHELXL97* (Sheldrick, 2008), Fc^*^=kFc[1+0.001xFc^2^λ^3^/sin(2θ)]^-1/4^
Primary atom site location: structure-invariant direct methods	Extinction coefficient: 0.00084 (18)

### Special details

**Table:**

Geometry. All e.s.d.'s (except the e.s.d. in the dihedral angle between two l.s. planes) are estimated using the full covariance matrix. The cell e.s.d.'s are taken into account individually in the estimation of e.s.d.'s in distances, angles and torsion angles; correlations between e.s.d.'s in cell parameters are only used when they are defined by crystal symmetry. An approximate (isotropic) treatment of cell e.s.d.'s is used for estimating e.s.d.'s involving l.s. planes.
Refinement. Refinement of *F*^2^ against ALL reflections. The weighted *R*-factor *wR* and goodness of fit *S* are based on *F*^2^, conventional *R*-factors *R* are based on *F*, with *F* set to zero for negative *F*^2^. The threshold expression of *F*^2^ > σ(*F*^2^) is used only for calculating *R*-factors(gt) *etc*. and is not relevant to the choice of reflections for refinement. *R*-factors based on *F*^2^ are statistically about twice as large as those based on *F*, and *R*- factors based on ALL data will be even larger.

### Fractional atomic coordinates and isotropic or equivalent isotropic displacement parameters (Å^2^)

**Table:**

	*x*	*y*	*z*	*U*_iso_*/*U*_eq_	
Cl1	0.14870 (5)	0.28558 (6)	0.06873 (6)	0.0950 (4)	
O1	0.24702 (14)	0.38109 (14)	0.46884 (14)	0.0866 (7)	
O2	0.17681 (13)	0.29247 (14)	0.49968 (15)	0.0980 (8)	
O3	0.36910 (10)	0.56885 (10)	0.18178 (11)	0.0646 (5)	
N1	0.29514 (11)	0.44612 (12)	0.16009 (13)	0.0507 (5)	
N2	0.33431 (12)	0.47740 (12)	0.09354 (12)	0.0520 (6)	
N3	0.50947 (13)	0.62326 (13)	−0.09159 (15)	0.0627 (6)	
N4	0.20600 (15)	0.33277 (15)	0.44731 (16)	0.0674 (7)	
C1	0.21758 (13)	0.35202 (14)	0.20311 (17)	0.0497 (6)	
C2	0.16520 (15)	0.30249 (16)	0.17913 (19)	0.0624 (8)	
C3	0.12506 (16)	0.26504 (17)	0.2413 (3)	0.0767 (10)	
H3	0.0893	0.2333	0.2231	0.092*	
C4	0.13799 (16)	0.27471 (16)	0.3291 (2)	0.0723 (9)	
H4	0.1120	0.2494	0.3713	0.087*	
C5	0.19049 (14)	0.32300 (14)	0.35332 (18)	0.0540 (7)	
C6	0.22899 (13)	0.36163 (14)	0.29285 (16)	0.0505 (6)	
H6	0.2632	0.3947	0.3120	0.061*	
C7	0.25958 (14)	0.39094 (15)	0.13746 (17)	0.0544 (7)	
H7	0.2603	0.3753	0.0793	0.065*	
C8	0.37206 (13)	0.53777 (14)	0.11106 (15)	0.0471 (6)	
C9	0.41915 (13)	0.56487 (13)	0.03806 (15)	0.0446 (6)	
C10	0.41588 (15)	0.54180 (14)	−0.04791 (15)	0.0543 (7)	
H10	0.3836	0.5060	−0.0645	0.065*	
C11	0.46139 (17)	0.57273 (16)	−0.10915 (17)	0.0643 (8)	
H11	0.4580	0.5568	−0.1671	0.077*	
C12	0.51235 (15)	0.64389 (16)	−0.00807 (19)	0.0632 (8)	
H12	0.5459	0.6789	0.0070	0.076*	
C13	0.46919 (15)	0.61719 (15)	0.05767 (17)	0.0588 (7)	
H13	0.4737	0.6343	0.1150	0.071*	
H2	0.3334 (16)	0.4566 (14)	0.0406 (10)	0.080*	

### Atomic displacement parameters (Å^2^)

**Table:**

	*U*^11^	*U*^22^	*U*^33^	*U*^12^	*U*^13^	*U*^23^
Cl1	0.0684 (6)	0.1253 (8)	0.0914 (6)	0.0108 (5)	−0.0135 (4)	−0.0546 (6)
O1	0.1009 (18)	0.1033 (19)	0.0558 (13)	−0.0094 (15)	−0.0011 (12)	0.0119 (12)
O2	0.1027 (18)	0.1125 (19)	0.0787 (15)	0.0079 (15)	0.0333 (13)	0.0459 (14)
O3	0.0837 (14)	0.0749 (13)	0.0351 (10)	−0.0072 (11)	0.0146 (9)	−0.0096 (9)
N1	0.0517 (13)	0.0618 (14)	0.0385 (11)	0.0045 (11)	0.0075 (10)	0.0043 (10)
N2	0.0645 (14)	0.0606 (15)	0.0308 (11)	0.0020 (12)	0.0090 (10)	−0.0004 (10)
N3	0.0636 (16)	0.0770 (17)	0.0476 (15)	0.0023 (13)	0.0087 (11)	0.0066 (12)
N4	0.0682 (17)	0.0753 (18)	0.0588 (16)	0.0173 (15)	0.0191 (13)	0.0226 (14)
C1	0.0445 (15)	0.0519 (16)	0.0526 (15)	0.0061 (12)	0.0028 (12)	−0.0037 (12)
C2	0.0493 (17)	0.0646 (19)	0.073 (2)	0.0108 (14)	0.0018 (15)	−0.0201 (15)
C3	0.0525 (19)	0.062 (2)	0.116 (3)	−0.0120 (15)	0.0152 (19)	−0.0258 (19)
C4	0.064 (2)	0.0575 (19)	0.095 (3)	−0.0031 (16)	0.0257 (18)	−0.0006 (17)
C5	0.0489 (16)	0.0497 (16)	0.0635 (18)	0.0065 (13)	0.0140 (13)	0.0059 (13)
C6	0.0469 (15)	0.0528 (16)	0.0519 (16)	0.0001 (12)	0.0037 (12)	0.0029 (12)
C7	0.0588 (17)	0.0681 (19)	0.0364 (14)	0.0076 (15)	0.0028 (12)	−0.0043 (13)
C8	0.0546 (16)	0.0541 (16)	0.0325 (13)	0.0091 (13)	0.0035 (11)	0.0015 (11)
C9	0.0470 (15)	0.0507 (15)	0.0359 (13)	0.0129 (12)	0.0034 (11)	0.0023 (11)
C10	0.0679 (18)	0.0570 (16)	0.0378 (14)	−0.0026 (14)	0.0091 (12)	−0.0018 (12)
C11	0.081 (2)	0.074 (2)	0.0377 (15)	0.0014 (18)	0.0124 (14)	−0.0056 (14)
C12	0.0542 (17)	0.078 (2)	0.0572 (18)	−0.0075 (15)	−0.0030 (14)	0.0078 (15)
C13	0.0644 (18)	0.0738 (19)	0.0383 (14)	−0.0027 (15)	−0.0019 (13)	−0.0018 (13)

### Geometric parameters (Å, °)

**Table:**

Cl1—C2	1.733 (3)	C3—H3	0.9300
O1—N4	1.222 (3)	C4—C5	1.376 (4)
O2—N4	1.220 (3)	C4—H4	0.9300
O3—C8	1.221 (3)	C5—C6	1.368 (3)
N1—C7	1.268 (3)	C6—H6	0.9300
N1—N2	1.374 (3)	C7—H7	0.9300
N2—C8	1.350 (3)	C8—C9	1.499 (3)
N2—H2	0.892 (10)	C9—C10	1.375 (3)
N3—C11	1.324 (4)	C9—C13	1.378 (3)
N3—C12	1.326 (3)	C10—C11	1.382 (4)
N4—C5	1.467 (4)	C10—H10	0.9300
C1—C2	1.388 (4)	C11—H11	0.9300
C1—C6	1.391 (3)	C12—C13	1.373 (4)
C1—C7	1.458 (4)	C12—H12	0.9300
C2—C3	1.390 (4)	C13—H13	0.9300
C3—C4	1.367 (4)		
			
C7—N1—N2	114.8 (2)	C5—C6—H6	119.6
C8—N2—N1	118.8 (2)	C1—C6—H6	119.6
C8—N2—H2	123 (2)	N1—C7—C1	119.7 (2)
N1—N2—H2	118 (2)	N1—C7—H7	120.2
C11—N3—C12	115.2 (2)	C1—C7—H7	120.2
O2—N4—O1	123.7 (3)	O3—C8—N2	122.9 (2)
O2—N4—C5	118.1 (3)	O3—C8—C9	121.2 (2)
O1—N4—C5	118.3 (2)	N2—C8—C9	115.9 (2)
C2—C1—C6	116.7 (2)	C10—C9—C13	117.1 (2)
C2—C1—C7	121.7 (3)	C10—C9—C8	124.8 (2)
C6—C1—C7	121.6 (2)	C13—C9—C8	118.1 (2)
C1—C2—C3	122.0 (3)	C9—C10—C11	118.8 (3)
C1—C2—Cl1	119.9 (2)	C9—C10—H10	120.6
C3—C2—Cl1	118.1 (2)	C11—C10—H10	120.6
C4—C3—C2	120.1 (3)	N3—C11—C10	124.9 (3)
C4—C3—H3	119.9	N3—C11—H11	117.5
C2—C3—H3	119.9	C10—C11—H11	117.5
C3—C4—C5	118.1 (3)	N3—C12—C13	124.6 (3)
C3—C4—H4	120.9	N3—C12—H12	117.7
C5—C4—H4	120.9	C13—C12—H12	117.7
C6—C5—C4	122.3 (3)	C12—C13—C9	119.5 (2)
C6—C5—N4	119.0 (3)	C12—C13—H13	120.3
C4—C5—N4	118.7 (3)	C9—C13—H13	120.3
C5—C6—C1	120.7 (3)		

### Hydrogen-bond geometry (Å, °)

**Table:**

*D*—H···*A*	*D*—H	H···*A*	*D*···*A*	*D*—H···*A*
N2—H2···O3^i^	0.89 (1)	2.19 (1)	3.055 (3)	164 (3)

Symmetry codes: (i) *y*−1/4, −*x*+3/4, *z*−1/4.
